# The Influence of Personality Traits on School Bullying: A Moderated Mediation Model

**DOI:** 10.3389/fpsyg.2021.650070

**Published:** 2021-05-21

**Authors:** Yun Zhang, Zuoshan Li, Yalan Tan, Xi Zhang, Qingyu Zhao, Xin Chen

**Affiliations:** ^1^Key Laboratory of Applied Psychology, Chongqing Normal University, Chongqing, China; ^2^School of Education, Chongqing Normal University, Chongqing, China

**Keywords:** school bullying, personality traits, loneliness, self-concept, adolescents

## Abstract

We recruited 1,631 middle and high school students to explore the relationship between personality traits and school bullying, and the moderated and mediating roles of self-concept and loneliness on this relationship. Results showed that (1) neuroticism had a significant positive predictive effect on being bullied, extroversion had a significant negative predictive effect on being bullied, and agreeableness had a significant negative predictive effect on bullying/being bullied; (2) loneliness played a mediating role between neuroticism and bullied behaviors, extroversion and bullying behaviors, and agreeableness and bullying/bullied behaviors; (3) self-concept played a moderating role on the mediation pathway of loneliness on neuroticism, extraversion, agreeableness and bullying behaviors. Therefore, to reduce the frequency of school bullying among adolescents, we should not only reduce their levels of loneliness but also improve their levels of self-concept.

## Introduction

School bullying has been defined as “a specific form of aggression, which is intentional, repeated, and involves a disparity of power between the victims and perpetrators” (Olweus, 1993a,b). In addition, some studies found that sexual violence (McMahon et al., 2019; Madrid et al., 2020) and cyberbullying (Livazović and Ham, 2019; Ige, 2020) were two emerging forms of adolescent violence in today’s society. Bullying is an extremely damaging type of violence present in schools all over the world (Zych et al., 2019). According to previous research, in addition to the effects of physical injury, bullying can lead to decreased self-confidence, self-esteem, and academic performance. It will cause inattention, absenteeism, anxiety, headaches, insomnia, nightmares, depression, and other related symptoms. In extreme cases, students may even commit suicide (Sharp and Smith, 1994). Due to the prefrontal cortex’s inhibition of physical activities, emotional processing is regulated by threat experience, and thus adolescents risk emotional dysregulation and increased internalization problems when they are subjected to threat experiences (Weissman et al., 2019). School problems, peer victimization, parent–child relationship quality issues, and friendship quality issues all affect the anxiety level of adolescents (Nelemans et al., 2017). Campus bullying cannot only cause depression in teenagers but it can also have a serious impact on their future social ability, learning ability, and academic performance (Chen and Chen, 2020). Bullying usually occurs in elementary school, a critical period in child development (Behnsen et al., 2020), and traditional bullying and cyberbullying victimization increase the likelihood of avoidance behaviors and of bringing a weapon to school (Keith, 2018). An alarming fact is that bullying can lead to antisocial behavior in adulthood (Stubbs-Richardson et al., 2018). Olweus (1993a, b) pointed out that, often, students who bully others have a higher individual crime rate when they grow up, almost four times higher than others. In addition, victims of bullying are more likely to commit crimes in the future (Behnsen et al., 2020). Studies have shown that bullying victimization and perpetration correlate strongly and that their cross-lagged longitudinal relationship runs in both directions, meaning that perpetration is just as likely to lead to future victimization as victimization is to lead to future perpetration (Walters, 2020). Individuals who experienced the vicarious form (peer victimization) had a higher likelihood of experiencing the same type of victimization as their peers (Stubbs-Richardson and May, 2020). Therefore, improving research on school bullying and its influencing factors is of great significance to the prevention and governance of school bullying.

Bullying behaviors were not effectively measured by demographic variable (Abuhammad et al., 2020). But there are many factors affecting school bullying, among which the influence of personality traits is undeniable. Personality was first studied by Alport. Cattell later identified 16 personality traits. In 1949, Fiske analyzed 22 personality traits from Cattell’s vocabulary and found that five factors always appeared first on the list. These factors came to be known as the Big Five: Openness (imaginative, aesthetic, emotional, unconventional, creative, intelligent, etc.); Conscientiousness (showing competence, fairness; being methodical and dutiful; achieving self-discipline, prudence, restraint, etc.); Extraversion (showing warmth, sociability, assertiveness, optimism, etc.; engaging in activities; risk-taking); Agreeableness (having the characteristics of trustworthiness, altruism, frankness, compliance, modesty, empathy, etc.); Neuroticism (experiencing anxiety, hostility, depression, self-awareness, impulsivity, vulnerability, inability to maintain emotional stability) (Peng, 2001).

Since then, many scholars have studied the relationship between school bullying and personality traits. The compensation model of aggression proposes that low self-esteem leads to bullying behaviors (Staub, 1989). Moreover, the model modified by Nail et al. (2016) proposes that a defensive personality structure is an essential factor in causing bullying. This model details how bullying behaviors are driven by a bully’s personality, motivations, including narcissism, defensive self-centeredness, and inconsistent levels of high self-esteem (Nail et al., 2016). Previous research has indicated that adolescents who have these personality characteristics are likely to be associated with school bullying (Thomaes et al., 2009; Simon et al., 2016). More specifically, personality traits, such as extraversion, conscientiousness, and neuroticism, are significantly associated with school bullying (Miao, 2019).

Extroversion and conscientiousness are negatively related to school bullying (Yao, 2017). Perpetrators of bullying have been found to be prone to anger, silence, and emotional sensitivity, as well as high self-evaluations and psychoticism (Zhou and Ding, 2003), demonstrating that emotional instability is one factor affecting school bullying. In addition, Gu and Zhang (2003) found that self-esteem, extroversion, and neuroticism can significantly predict bullying or being bullied, and self-esteem, psychoticism, and neuroticism are significantly related to bullying in their study of the relationship between the bullying behaviors and personality traits of students in primary schools. Moreover, results of the independent analysis of the victims and perpetrators found that for perpetrators, their personalities are as a whole, and their bullying behaviors were in most cases caused by the interaction of negative cognitive tendencies towards society, negative attitudes toward bullying events, hyperactivity, emotional temperament characteristics, and specific stimulus events. And for victims, school bullying may be harmful to their personality development, and being bullied may also be associated with their own personality traits (Zhang et al., 2001).

Even though personality traits have been shown to have a significant impact on school bullying, there is no evidence to date demonstrating that personality traits can directly affect school bullying behaviors. The social bonds theory (Hirschi, 1969) notes that the links between increased crime rates and individuals and society are weak, whereas increased crime rates are closely related to a low consistency in social norms. In the study by Zhang et al. (2016), personality was significantly correlated with loneliness, and agreeableness, extroversion, openness, and conscientiousness were significantly negatively correlated with loneliness. According to Costa and Mccrae (1992), loneliness is an unpleasant experience that occurs when individuals feel that their social, interpersonal network is low in quality or insufficient in quantity. Furthermore, Zhang (2019) found that school bullying behaviors are significantly correlated with loneliness in elementary school students. School bullying and being bullied are also positively correlated with loneliness in middle school students (Zhou and Ding, 2003). It can therefore be seen that personality traits might affect loneliness, and loneliness might influence school bullying. Therefore, we proposed hypothesis one: loneliness plays a mediating role in the relationship between personality traits and school bullying.

Self-concept is defined by Shavelson as an individual’s overall view of himself based on interpersonal communications and living environment (Byrne and Shavelson, 1996). Previous research has also shown that various dimensions of personality traits have significant relationships with self-concept. For example, self-concept is highly positively correlated with extroversion, conscientiousness, and agreeableness, and it has a moderate positive correlation with openness and moderately negative correlation with neuroticism (Xiang et al., 2006). In the study by Xiang et al. (2006), personality was quickly clustered by researchers into categories 3–6. The results showed that four categories were justifiable: harmonious personalities (low scores for neuroticism and high scores for other dimensions); emotional personalities (very unstable neuroticism scores, medium scores for other dimensions); conservative personalities (low scores in all dimensions); passive personalities (average scores for neuroticism, low scores for all other dimensions). Furthermore, there are significant differences in the levels of self-concept in students who have different personality traits. Specifically, students with harmonious personalities have the highest levels of self-concept, followed by those with conservative personalities and finally, passive personalities which have the lowest (Xiang et al., 2006). Children’s self-concepts also play mediating roles in the influences of peer rejection and offensive behaviors on children’s relational aggression and physical attacks (Ji et al., 2012). The self-concept and self-esteem of adolescents are closely related to problem behaviors, and adolescents may attack others because their self-concepts are low (Donnellan et al., 2005; Diamantopoulou et al., 2008) or when they perceive that others do not recognize their self-concepts (Taylor et al., 2007; Diamantopoulou et al., 2008). With regard to this phenomenon, humanistic psychology explains that negative self-attention and vague self-concepts result in aggressive behaviors (Donnellan et al., 2005). It is easy to see that the level of self-concept is not only related to personality traits but also affects the adolescents’ being bullied and the bullying behaviors of perpetrators. In addition, the clarity of adolescents’ self-concept is significantly negatively correlated with loneliness (Xu et al., 2017). Students who have lower self-concepts suffer strong feelings of loneliness (Chen and Zhang, 2010), meaning that individuals with weaker self-concepts tend to develop high levels of loneliness.

In summary, personality traits significantly influence school bullying and loneliness, and loneliness also affects school bullying. Students with weaker self-concepts tend to develop high levels of loneliness, and high levels of loneliness may predict aggressive behaviors. Therefore, different self-concept levels could affect the development of loneliness, while the degree of loneliness could affect the impact of personality traits on school bullying. Thus, we proposed hypothesis two: self-concept plays a moderating role in the mediation pathway for loneliness on the relationship between personality traits and school bullying.

**FIGURE 1:**
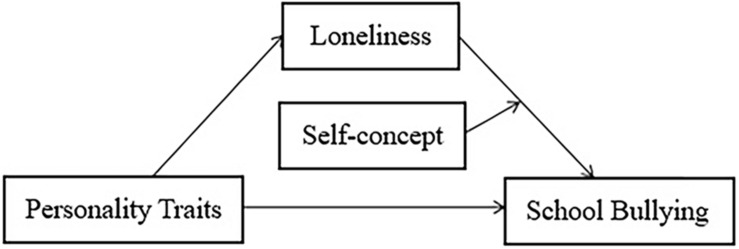
The moderated mediating model.

The purpose of the current study was to establish a moderated mediation model (see Figure 1) to explore the mediating role of loneliness on the relationship between personality traits and school bullying, as well as the moderating role of self-concept in the mediation pathway.

## Materials and Methods

### Participants

A total of 2,000 adolescents at two high schools in Chongqing and Shandong received the questionnaire survey through convenience sampling. On completion, 1,631 valid questionnaires were returned reflecting an effective response rate of 81.55%.

Participants were aged 11–21 years old [mean (*M*) ± standard deviation (*SD*) = 15.39 ± 1.37], with 755 (46.3%) being male and 876 (53.7%) being female. Among the junior high school students, 88 were from the first grade, 99 were from the second grade, and 275 were from the third grade. Among the senior high school students, 606 were from the first grade, 522 were from the second grade, and 47 were from the third grade.

### Questionnaires

#### NEO Five-Factor Inventory (NEO-FFI)

We used the NEO-FFI Questionnaire which was modified by Costa and Mccrae (1992). There are 60 questions which constitute five subscales: neuroticism, extraversion, openness, agreeableness, and conscientiousness. The α coefficient in this study was 0.70.

#### Chinese Version of Bully/Victim Questionnaire for Middle Students (BVQ-C)

We used the Bully/Victim Questionnaire established by Olweus (1993a, b) and modified by Zhang and Wu (1999). The α coefficient in this study was 0.903.

#### Self-Concept Clarity Scale (SCC)

We used the Self-concept Clarity Scale established by Campbell et al. (1996) and modified by Chen and Ouyang (2013). There are 12 questions in total (including “My view of myself often conflicts with other people’s view of me,” “My thoughts about myself change very frequently,” etc.). Use a 5-point Likert score (1 = “strongly disagree,” 5 = “strongly agree”) to evaluate, and calculate the total score of all questions in the scale. This scale effectively reflects the extent to which individuals clearly determine their own self-concept. The α coefficient in this study was 0.758.

#### UCLA Loneliness Scale

We used the UCLA Loneliness Scale established by Russell (1996) and modified by Wang (1995). There are 18 questions in total. The scale consists of 18 items (including “I feel sorry for others,” “I feel so lonely,” “I cannot find someone I can talk to,” etc.) using a 4-point score from “never” to “always.” The α coefficient in this study was 0.892.

### Data Collection and Analysis

The self-reported questionnaire was completed anonymously during school classes. The researchers were postgraduate students in the Key Laboratory of Applied Psychology, proficient in psychological research methods. Data collection was completed in February 2020. Statistical analysis was performed using SPSS 18.0. Our research has been registered on the Open Science Framework. https://osf.io/8x6a4/.

## Results

### Control and Inspection of Common Method Biases

In this study, data were obtained from questionnaires meaning that common method biases might affect the results. In order to minimize these influences, we adopted control measures, such as reverse scoring, anonymous reporting, and Harman’s single factor test (Podsakoff et al., 2003). Results showed 21 factors with characteristic roots over 1, and the variance explanation rate of the first common factor was 14.62%, which was less than 40%. Therefore, the current study was not significantly affected by common method biases, and the data were deemed reliable.

### Descriptive Statistics and Correlation Analysis

Our study performed descriptive statistics and correlation analysis on the five personality trait dimensions, self-concept, loneliness, and the two dimensions of school bullying (bullying/being bullied). We found that neuroticism was significantly positively correlated with self-concept, loneliness, and being bullied. Extraversion was significantly negatively correlated with self-concept, loneliness, and being bullied. Openness was significantly negatively correlated with self-concept and loneliness. Agreeableness was significantly negatively correlated with self-concept, loneliness, and bullying/being bullied. Loneliness was significantly positively correlated with self-concept and bullying/being bullied. Self-concept and being bullied were significantly positively correlated. However, in our study, openness and conscientiousness were not significantly correlated with bullying/being bullied. In some western studies, teenagers who reported bullying scored lower on conscientiousness and openness (Turner and Ireland, 2010; Fossati et al., 2012), as well as lower level of conscientiousness was also associated with being bullied (Effrosyni and Theodoros, 2015). Different from the western countries, children in the traditional Chinese families are not truly independent until they get married, and before that, they may live under the control of their parents, thus there may be ideological differences on conscientiousness and openness. Additionally, in our other interview study, we found that few class leaders with good grades and strong sense of responsibility also have some bullying behaviors, such as verbal bullying and relationship manipulation. So we suspect that conscientiousness and openness did not affect school bullying directly in this study probably because of regional and cultural differences, as well as the selection of samples. The specific results are shown in Table 1.

**TABLE 1:** Descriptive statistics and correlation coefficients for each variable (*N* = 1,631).

	*M*	*SD*	1	2	3	4	5	6	7	8	9
1 Neuroticism	34.11	8.66	1								
2 Extraversion	39.77	7.63	−0.50**	1							
3 Openness	39.28	5.62	−0.21**	0.35**	1						
4 Agreeableness	43.34	5.63	−0.39**	0.33**	0.34**	1					
5 Conscientiousness	36.94	6.33	−0.10**	0.37**	0.47**	0.26**	1				
6 Loneliness	38.17	10.03	0.58**	−0.63**	−0.32**	−0.42**	−0.26**	1			
7 Self-concept	36.50	7.42	0.52**	−0.26**	−0.15**	−0.28**	−0.031	0.49**	1		
8 Being bullied	7.42	3.37	0.20**	−0.13**	−0.04	−0.23**	0.004	0.28**	0.12**	1	
9 Bullying	6.63	2.65	0.026	−0.034	−0.026	−0.15**	0.013	0.12**	−0.008	0.55**	1

*p < 0.05, **p < 0.01, ***p < 0.001.

### Influence of Personality Traits on School Bullying: Test of the Moderated Mediating Model

First, the data were standardized. Second, the macro program PROCESS in SPSS was used to test the moderated mediating model. Finally, the deviation correction and the percentile Bootstrap method were set. The number of repeated sampling was set to 5,000 for inspection and the confidence interval (CI) was set to 95%. The results are shown in Table 2.

**TABLE 2:** Test of the moderated mediating model of loneliness.

Regression equation (*N* = 1,631)		Fit index			Significance	
Outcome Variable	Predictive variable	*R*	*R*^2^	*F*	β	*t*
Loneliness		0.603	0.363	309.39		
	Neuroticism				0.566	28.025***
Being bullied		0.248	0.061	35.457		
	Neuroticism				0.216	8.804***
Being bullied		0.313	0.098	44.277		
	Neuroticism				0.08	2.722**
	Loneliness				0.241	8.152***
Loneliness		0.635	0.402	365.685		
	Extraversion				−0.619	−30.739***
Being bullied		0.178	0.032	17.819		
	Extraversion				−0.129	−5.047***
Being bullied		0.312	0.097	43.865		
	Extraversion				0.076	2.432**
	Loneliness				0.331	10.870***
Loneliness		0.447	0.2	135.74		
	Agreeableness				−0.389	−17.134***
Being bullied		0.253	0.065	37.374		
	Agreeableness				−0.224	−9.119***
Being bullied		0.313	0.098	44.277		
	Agreeableness				−0.133	−5.097***
	Loneliness				0.234	8.946***
Loneliness		0.419	0.176	346.908		
	Agreeableness				−0.419	−18.626***
Bullying		0.149	0.022	37.033		
	Agreeableness				−0.149	−6.086***
Bullying		0.162	0.026	21.949		
	Agreeableness				−0.120	−4.448***
	Loneliness				0.070	2.595**

**p < 0.01, ***p < 0.001.

The test of the mediating effect was then conducted. We used Model 4 of the SPSS macro designed by Hayes (2012) controlling for gender and age (not shown in the table) to perform the mediating effect test of loneliness on the various personality trait dimensions. Results of the regression analysis (see Tables 2, 3) showed that neuroticism had a positive predictive effect on being bullied, β = 0.216, *p* < 0.001. After incorporating loneliness into the regression equation, neuroticism still had a significantly predictive effect on being bullied, β = 0.080, *p* < 0.01, and a positively predictive effect on loneliness, β = 0.566, *p* < 0.001. Loneliness had a positively predictive effect on being bullied, β = 0.241, *p* < 0.001, Boot *SE* = 0.010, 95% CI = 0.062, 0.100. This indicated that the mediating effect of loneliness on the relationship between neuroticism and being bullied was significant.

**TABLE 3:** Decomposition of the total, direct and mediating effects.

Predictive variable		Effect Size	Boot *SE*	Boot LLCI	Boot ULCI	Ratio
Neuroticism	Total effect	0.084	0.010	0.065	0.103	
	Direct effect	0.031	0.011	0.009	0.053	36.98%
	Mediating effect	0.053	0.008	0.038	0.069	63.02%
Extraversion	Total effect	−0.057	0.011	−0.079	−0.035	
	Direct effect	0.034	0.014	0.007	0.061	26.98%
	Mediating effect	−0.091	0.011	−0.114	−0.070	73.02%
Agreeableness (Being bullied)	Total effect	−0.134	0.015	−0.163	−0.105	
	Direct effect	−0.079	0.016	−0.110	−0.049	59.30%
	Mediating effect	−0.055	0.008	−0.070	−0.040	40.70%
Agreeableness (Bullying)	Total effect	−0.149	0.025	−0.197	−0.101	
	Direct effect	−0.120	0.027	−0.173	−0.067	80.35%
	Mediating effect	−0.029	0.011	−0.051	−0.010	19.65%

*SE, standard error; LLCI, lower limit confidence interval; ULCI, upper limit confidence interval.*

Similarly, extraversion had a negatively predictive effect on being bullied, β = −0.129, *p* < 0.001. After incorporating loneliness into the regression equation, extroversion converted to a significantly positive predictive effect on being bullied, β = 0.076, *p* < 0.01, and a negatively predictive effect on loneliness β = −0.619, *p* < 0.001. Loneliness had a positively predictive effect on being bullied, β = 0.331, *p* < 0.001, Boot *SE* = 0.010, 95% CI = 0.091, 0.132. This showed that the mediating effect of loneliness on the relationship between extroversion and being bullied was significant.

Agreeableness had a negatively predictive effect on being bullied, β = −0.224, *p* < 0.001. After incorporating loneliness into the regression equation, agreeableness still had a significant predictive effect on being bullied, β = −0.133, *p* < 0.01, and a negatively predictive effect on loneliness, β = −0.42, *p* < 0.001. Loneliness had a positively predictive effect on being bullied β = 0.25, *p* < 0.001, Boot *SE* = 0.009, 95% CI = 0.062, 0.096. This showed that the mediating effect of loneliness on the relationship between agreeableness and being bullied was significant.

In addition, agreeableness had a negatively predictive effect on bullying, β = −0.149, *p* < 0.001. After incorporating loneliness into the regression equation, agreeableness still had a significantly predictive effect on bullying, β = −0.120, *p* < 0.001, and a negatively predictive effect on loneliness, β = −0.419, *p* < 0.001. Loneliness had a positively predictive effect on bullying, β = 0.070, *p* < 0.01, Boot *SE* = 0.027, 95% CI = 0.017, 0.123. This showed that the mediating effect of loneliness on the relationship between agreeableness and bullying was significant.

It should be noted that the upper and lower limits for the bootstrap 95% CIs for the direct effects of neuroticism, extroversion, and agreeableness on bullying/being bullied behaviors, as well as the mediating effect of loneliness, did not contain 0 (see Table 3). This showed that neuroticism, extroversion, and agreeableness could not only directly influence bullying/being bullied behaviors but also could predict bullying/being bullied behaviors through the mediating effect of loneliness.

The test of the moderated mediation model was then performed. We established a moderated mediation model consisting of three personality trait dimensions (neuroticism, extraversion, and agreeableness) and school bullying/being bullied, in which we regarded loneliness as the mediating variable and self-concept as the moderating variable using the Model 14 of the SPSS macro designed by Hayes (2012). The results are shown in Tables 4, 5.

**TABLE 4:** Test of the moderated mediating model.

Regression equation (*N* = 1,631)		Fit index			Significance	
Outcome Variable	Predictor Variable	*R*	*R*^2^	*F*	β	*t*
Being Bullied		0.323	0.1043	31.5195		
	Neuroticism				0.087	2.786**
	Loneliness				0.238	7.911***
	Self-concept				−0.001	−0.047
	Gender				−0.258	−5.379***
	Age				−0.017	−0.938
	Loneliness × Self-concept				0.064	3.293**
Being Bullied		0.3205	0.1027	30.9824		
	Extraversion				0.069	2.204*
	Loneliness				0.317	9.702***
	Self-concept				0.025	0.947
	Gender				−0.235	−4.937***
	Age				−0.007	−0.380
	Loneliness (Self-concept				0.059	3.051**
Being Bullied		0.3412	0.1164	35.6585		
	Agreeableness				−0.144	−5.487***
	Loneliness				0.227	8.069***
	Self-concept				0.009	0.350
	Gender				−0.215	−4.543***
	Age				−0.024	−1.377
	Loneliness × Self-concept				0.075	3.845***

*p < 0.05, **p < 0.01, ***p < 0.001.

**TABLE 5:** Direct and mediating effects for different levels of self-concept.

Predictive variable		Self-concept	Effect Size	Boot *SE*	Boot LLCI	Boot ULCI
Neuroticism	Direct effect	29.081 (*M* − 1 *SD*)	0.059	0.012	0.034	0.083
		36.49 (*M*)	0.080	0.010	0.060	0.100
		43.916 (*M* + 1 *SD*)	0.102	0.012	0.079	0.125
	Mediating effect of loneliness	29.081 (*M* − 1 *SD*)	0.038	0.009	0.020	0.057
		36.499 (*M*)	0.052	0.007	0.038	0.068
		43.916 (*M* + 1 *SD*)	0.067	0.012	0.044	0.090
Extraversion	Direct effect	29.081 (*M* − 1 *SD*)	0.087	0.013	0.061	0.113
		36.499 (*M*)	0.107	0.011	0.085	0.128
		43.916 (*M* + 1 *SD*)	0.127	0.012	0.102	0.151
	Mediating effect of loneliness	29.081 (*M* − 1 *SD*)	−0.071	0.013	−0.097	−0.044
		36.499 (*M*)	−0.087	0.011	−0.109	−0.065
		43.916 (*M* + 1 *SD*)	−0.103	0.015	−0.133	−0.074
Agreeableness	Direct effect	29.081 (*M* − 1 *SD*)	0.051	0.012	0.028	0.075
		36.499 (*M*)	0.076	0.009	0.058	0.095
		43.916 (*M* + 1 *SD*)	0.101	0.011	0.080	0.123
	Mediating effect of loneliness	29.081 (*M* − 1 *SD*)	−0.035	0.010	−0.057	−0.015
		36.499 (*M*)	−0.053	0.008	−0.068	−0.038
		43.916 (*M* + 1 *SD*)	−0.070	0.011	−0.094	−0.049

From Table 4, we can see that for the dimension of neuroticism, the product term of loneliness and self-concept had a significant predictive effect on being bullied, *t* = 3.293, *p* < 0.01, after incorporating self-concept into the model, which meant that self-concept played a moderated role in the predictive effect of loneliness on being bullied. Similarly, for the dimension of extroversion, the product term of loneliness and self-concept had a significant predictive effect on being bullied, *t* = 3.051, *p* < 0.01, as well as for the dimension of agreeableness, and the product term for loneliness and self-concept had a significant predictive effect on being bullied, *t* = 3.845, *p* < 0.001. Thus, self-concept played a moderating role in the predictive effect of loneliness on being bullied both for the dimension of extroversion and agreeableness. However, self-concept did not play a moderating role in the predictive effect of loneliness on school bullying for the dimension of agreeableness, so these results are not presented.

**FIGURE 2:**
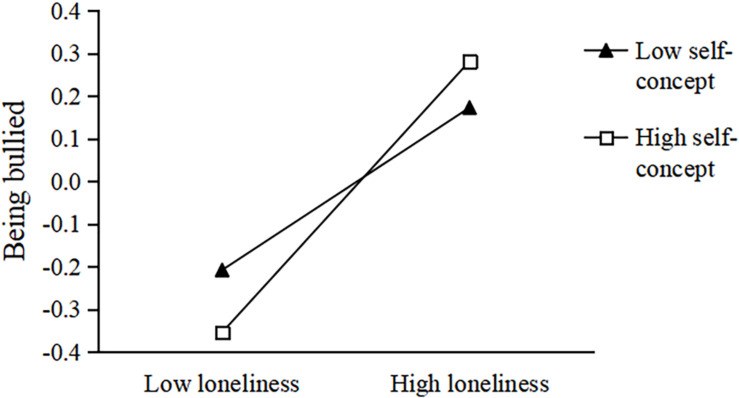
The influence of loneliness on the relationship between being bullied and neuroticism: the moderated effect of self-concept. The black triangle represents low self-concept and the white square represents high self-concept.

**FIGURE 3:**
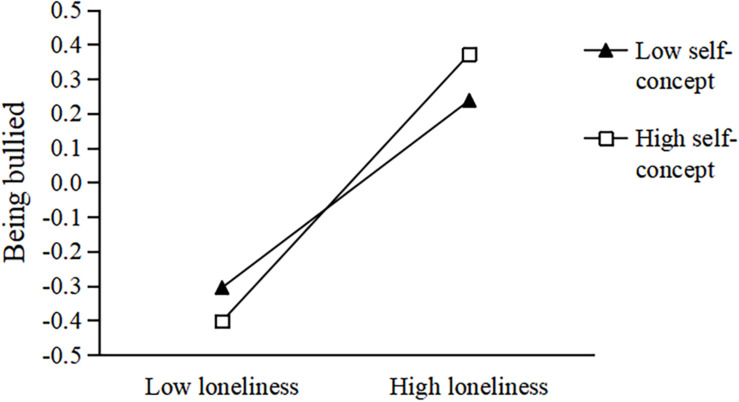
The influence of loneliness on the relationship between being bullied and extroversion: the moderated effect of self-concept. The black triangle represents low self-concept and the white square represents high self-concept.

**FIGURE 4:**
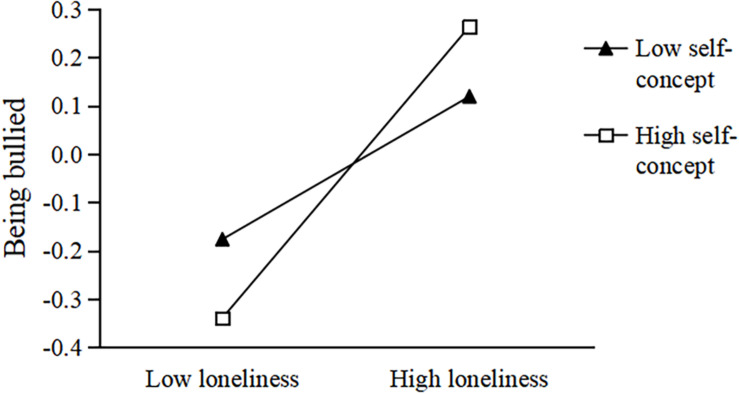
The influence of loneliness on the relationship between being bullied and agreeableness: the moderated effect of self-concept. The black triangle represents low self-concept and the white square represents high self-concept.

To better understand the moderated effect, we performed a simple slope test (Aiken and West, 1991). Data were divided into high and low groups according to self-concept values (*M* ± 1 *SD*). In the second half of the neuroticism-loneliness-being bullied pathway, when the level of self-concept was −1 *SD*, loneliness had a significant predictive effect on being bullied, *b* = 0.191, *t* = 5.335, *p* < 0.001, 95% CI = 0.195, 0.349. When the level of self-concept was +1 *SD*, loneliness still had a significant predictive effect on being bullied, *b* = 0.318, *t* = 9.130, *p* < 0.001, 95% CI = 0.250, 0.387 (see Figure 2). Meanwhile, in the second half of the extroversion-loneliness-being bullied pathway, when the level of self-concept was −1 *SD*, loneliness had a significant predictive effect on being bullied, *b* = 0.271, *t* = 6.917, *p* < 0.001, 95% CI = 0.195, 0.349. When the level of self-concept was +1 *SD*, loneliness still had a significant predictive effect on being bullied, *b* = 0.388, *t* = 10.519, *p* < 0.001, 95% CI = 0.315, 0.460 (see Figure 3). Moreover, in the second half of the agreeableness-loneliness-being bullied pathway, when the level of self-concept was −1 *SD*, loneliness had a significant predictive effect on being bullied, *b* = 0.148, *t* = 4.252, *p* < 0.001, 95% CI = 0.080, 0.216. When the level of self-concept was +1 *SD*, loneliness still had a significant predictive effect on being bullied, *b* = 0.302, *t* = 9.184, *p* < 0.001, 95% CI = 0.237, 0.366 (see Figure 4).

To sum up, when individuals had a low level of loneliness, the increased self-concept level was beneficial in reducing the occurrence of being bullied. However, when individuals had a high level of loneliness, the increased level of self-concept increased the occurrence of being bullied. Therefore, improving self-concept levels could reduce the incidence of being bullied by individuals with low levels of loneliness. Reducing the levels of loneliness would be conducive to reducing the incidence of being bullied by individuals with high loneliness.

## Discussion

The current study explored the direct impact of personality traits on school bullying and the mediating role of loneliness on the relationship between personality traits and school bullying/being bullied.

### Mediating Role of Middle School Students’ Loneliness

Results showed that loneliness played a mediating role in the relationships between neuroticism, extroversion, agreeableness, and being bullied. Therefore, each specific personality trait not only directly affected school bullying behaviors but also influenced being bullied behaviors through the mediating effect of loneliness.

When an individual’s interpersonal relationships do not reach their aspiration level, they are likely to experience loneliness, and this is accompanied by negative psychological states such as emptiness, boredom, helplessness, and depression (Kim, 2017). Personality is an essential influencing factor on loneliness (Schmitt and Kurdek, 1985). For example, extroversion, conscientiousness, and openness are positively related to adolescents’ effective social adaptation (Nie et al., 2008). Adolescents with high neuroticism levels generally have low interpersonal satisfaction (Lopes et al., 2003). In contrast, individuals with high agreeableness levels are better at controlling their emotions and resolving interpersonal conflicts (Graziano et al., 1996).

Individuals with a high level of extroversion are also better at communicating (Rothbart and Hwang, 2005), so they more easily establish positive interpersonal relationships. Therefore, personality traits affect the quality of interpersonal relationships, and aspiration levels of interpersonal relationships influence levels of loneliness, which can lead to school bullying/being bullied behaviors. To prevent teenagers from being negatively affected by school bullying/being bullied, it is necessary to cultivate good interpersonal relationships among adolescents and reduce their loneliness. In addition, studies indicated that a problematic parent–child relationship negatively predicted loneliness and depression in children (Fan and Wu, 2020), and the parent–child relationship could have a significant influence on school bullying. Some studies found that higher parental rejection and lower parental warmth predicted increases in peer victimization and vice versa (Kaufman et al., 2019). Research studies provided the evidence of highly significant effects of parenting interventions on bullying reduction (Chen et al., 2020). And some studies indicated that maternal love withdrawal prospectively predicted more aggressive bullying behaviors, whereas guilt induction predicted lower levels of aggressive bullying behaviors in children 6 months later (Yu et al., 2019). Therefore, bullying behaviors can be prevented by establishing a good parent–child relationship.

### Moderated Role of Middle School Students’ Self-Concept

After exploring the mediating role of loneliness on the relationship between personality traits and school bullying behaviors, we further examined the moderated role of self-concept in this mediating pathway.

The results showed that first, self-concept played a moderating role in the mediating pathway of loneliness on neuroticism and being bullied. Previous research has found that neuroticism is positively related to school bullying, and higher neuroticism is associated with a greater likelihood of psychological stress, impulsivity, and emotional reactivity (Zhou et al., 2019). On the contrary, teenagers with healthy mental states have a better understanding of all aspects of their self-concept, and their relationships in different domains (such as teacher–student, peer–peer, and parent–child relationships) are more harmonious (Shen et al., 2019). Crimes committed by young offenders may be related to their lack of a positive self-concept (Zhong and Liu, 2013), which means that adolescents with high self-concepts tend to have fewer problematic behaviors. Self-concept in the mediating effect of loneliness on the relationship between neuroticism and being bullied would also promote the healthy development of adolescents who are experiencing bullying dilemmas.

Second, the results showed that self-concept played a moderating role in the mediating effect of loneliness on the relationship between extroversion and being bullied. In our study, extroversion was significantly negatively related to bullying, showing that more introverted teenagers are more vulnerable to being bullied. Zhou et al. (2008) reported that individuals who are outgoing, cheerful, easy-going, and self-disciplined have higher levels of positive mental health, indicating that introversion and extroversion impact an individuals’ levels of mental health. The poorer the level of mental health, the more ambiguous the self-concept is, thus the less effective moderated effect of self-concept in the mediating role of loneliness on the relationship between extroversion and being bullied, leading to bullying behaviors. Therefore, enhancing students’ self-concept would be a macro measure, closely associated with parenting patterns, social supports (Li et al., 2017) and peer relationships (Li et al., 2013), as well as the differing developmental characteristics in every stage of teenagers’ growth. Due to the moderating role of self-concept in the mediating effect of loneliness on personality traits and bullying/being bullied, constructing comfortable school atmospheres (Xie and Mei, 2019) and perfecting personality educations (Miao, 2019) may overcome the negative impacts of school bullying. To be specific, first, education departments should offer targeted psychological guidance according to the different personality characteristics of teenagers, such as counseling for bullies with low self-esteem, paying attention to vulnerable victims with high self-esteem (Choi and Park, 2018), and preventing bullying by those with defensive personalities. Second, mental health courses on cultivating a healthy personality should be offered to teenagers. Such courses could strengthen self-affirmation training (Thomaes et al., 2009) and cultivate emotional regulation ability (Garofalo et al., 2016). Studies showed that educational interventions are effective in reducing the frequency of traditional and cyberbullying victimization and perpetration (Ng et al., 2020).

Third, our results showed that self-concept played a moderating role in the mediating pathway of loneliness on the relationship between agreeableness and being bullied, but it had no moderating role in the mediating effect of loneliness between agreeableness and bullying. The simple slope test results found that when school bullying occurred, there was a more obvious moderated effect of self-concept on students with low loneliness. Consequently, the levels of self-concept had a more significant effect on bullying. In the present study, agreeableness and bullying/being bullied were both significantly negatively correlated. One previous study manifested that agreeableness has a significantly negative correlation with depressive symptoms. Specifically, adolescents who are friendly and obedient are more likely to be approved by parents and society, and thus they are less likely to encounter adverse life events and have relatively fewer depressive symptoms (Zhang et al., 2019). Moreover, being bullied is closely related to depression (Chen et al., 2020). Being bullied could increase the severity of students’ depression (Cao et al., 2020), which would reduce their agreeableness level. Hence, higher agreeableness is associated with lower levels of bullying/being bullied, especially for adolescents who get along well with their classmates and teachers and experience harmonious family atmospheres.

Why did self-concept have a significant moderated effect on being bullied but no significant moderated effect on bullying? Scores for neuroticism increase with age, meaning that scores for some other personality traits may be replaced by it over time (Zhang et al., 2019). In addition, when individuals are in the transition from childhood to adolescence, personality traits might temporarily err towards immaturity. There is a temporary decline in the level of agreeableness from late childhood to middle adolescence (Akker et al., 2014). Consequently, a decline in agreeableness may explain why the moderated effect of self-concept on the mediating pathway was not significant in our study. Specific factors need to be further studied.

To sum up, school bullying/being bullied is harmful to students’ physical and mental health, so this issue deserves our continuous attention and reflection. Levels of neuroticism, openness, and agreeableness can positively or negatively predict school bullying. Furthermore, a high level of loneliness could exacerbate bullying/being bullied, while a higher self-concept could reduce the incidence of school bullying. Therefore, helping students have an unambiguous self-concept as well as reducing their loneliness are crucial approaches to reducing school bullying. However, some studies indicate that existing educational interventions had a very small to zero effect size on traditional bullying and cyberbullying perpetration. More research is needed to identify the key moderators that enhance educational programs and to develop alternative forms of anti-bullying interventions (Ng et al., 2020). Additionally, bullying can also be caused by some unconventional factors nowadays, such as the long-term use of adult drugs (alcohol, tobacco, various drugs; Zych et al., 2021) and dating violence (Quinn and Stewart, 2018). Thus, this situation implies that educators’ responses to school bullying should adapt to the rapidly changing modern world.

## Conclusion

Our key findings can be summarized as follows:

1) Neuroticism had a significantly positive predictive effect on being bullied, extroversion had a significantly negative predictive effect on being bullied, and agreeableness had a significantly negative predictive effect on bullying/being bullied.
2) Loneliness played a mediating role between neuroticism and bullied behaviors, extroversion and bullying behaviors, and agreeableness and bullying/bullied behaviors.
3) Self-concept played a moderated role in the mediation pathway of loneliness in neuroticism, extraversion, agreeableness, and bullying behaviors.

## Data Availability Statement

The datasets presented in this study can be found in online repositories. The names of the repository/repositories and accession number(s) can be found below: https://pan.baidu.com/s/1yzvKsezYCG8ihuKmSTIcWA; password: nyh8.

## Ethics Statement

The studies involving human participants were reviewed and approved by the Local Research Ethics Committee of Chongqing Normal University. Written informed consent to participate in this study was provided by the participants’ legal guardian/next of kin.

## Author Contributions

YZ: the principal author of the manuscript, consult literature, and logging date. ZL: advisor. YT: data analysis. XZ, QZ, and XC: logging date. All authors contributed to the article and approved the submitted version.

## Conflict of Interest

The authors declare that the research was conducted in the absence of any commercial or financial relationships that could be construed as a potential conflict of interest.
